# ALCOHOL CONSUMPTION AMONG ADULTS IN WISCONSIN

**DOI:** 10.1093/geroni/igad104.2513

**Published:** 2023-12-21

**Authors:** Amy Knepple Carney, Imagine Manders, Allison Hammond, Leslie Wessel

**Affiliations:** University of Wisconsin- Oshkosh, Oshkosh, Wisconsin, United States; University of Wisconsin-Oshkosh, Oshkosh, Wisconsin, United States; University of Wisconsin-Oshkosh, Oshkosh, Wisconsin, United States; University of Wisconsin-Oshkosh, Oshkosh, Wisconsin, United States

## Abstract

Alcohol usage as we age can lead to worsening health conditions and higher rates of accidents. There have been mixed results on alcohol use in adulthood, and many of these studies use only one metric of alcohol consumption. With higher-than-average (55.1%) drinking rates in the United States, Wisconsin currently ranks third highest for adult alcohol consumption (64.4%), it is essential to understand age trends for nondrinking, binge drinking, and heavy drinking. Using 2021 data from the CDC, we examined a series of logistic regressions to determine age differences in alcohol consumption. First, whether a person had at least one drink in the past 30 days, odds ratios show that relative to older adults (65+), 45-to-64-year-olds are 1.35 times more likely, 25-to-44-year-olds are 1.72 times more likely, and 18–to-24-year-olds are 1.09 times more likely to have reported having at least one drink in the last 30 days. Second, binge drinking, odds ratios show that relative to older adults (65+), 45-to-64-year-olds are 2.95 more likely, 25-to-44-year-olds are 5.41 times more likely, and 18-to-24-year-olds are 3.86 times more likely to binge drink. Last, heavy drinking, odds ratios show that relative to older adults (65+), 45-to-64-year-olds are 1.84 more likely, 25-to-44-year-olds are 1.94 times more likely, and 18-to-24-year-olds are 1.71 times more likely to drink heavily. Implications of these results are examined more fully but include the adverse effects on health for young-to-middle-aged adults, the potential to die younger for heavy drinkers, and the positive effects of lower drinking rates in later adulthood.

